# A trans-disciplinary approach to assessing police responses to mental health crisis: development of the de-escalating persons in crisis competencies tool (DePICT)

**DOI:** 10.3389/fpsyg.2025.1586502

**Published:** 2025-09-10

**Authors:** Jennifer A. A. Lavoie, Natalie Álvarez, Krystle Martin, Michael Girard, Terry G. Coleman

**Affiliations:** 1 Departments of Criminology and Psychology, Faculty of Human and Social Sciences, Wilfrid Laurier University, Brantford, ON, Canada; 2 Centre for Public Safety and Well-Being, Wilfrid Laurier University, Brantford, ON, Canada; 3 Centre for Research on Security Practices, Wilfrid Laurier University, Brantford, ON, Canada; 4 Scholarly, Research and Creative Activities, The Creative School, Toronto Metropolitan University, Toronto, ON, Canada; 5 Theatre and Performance Studies, The Creative School, Toronto Metropolitan University, Toronto, ON, Canada; 6 Dr. Krystle Martin and Associates, Durham, ON, Canada; 7 Health Sciences, Social Sciences and Humanities, Ontario Tech University, Oshawa, ON, Canada; 8 York University, Toronto, ON, Canada; 9 Ontario Police College, Aylmer, ON, Canada; 10 Moose Jaw Police Service, Moose Jaw, SK, Canada

**Keywords:** de-escalation, mental health crisis, competency, police training, relational policing, police reform, assessment

## Abstract

**Introduction:**

Police interactions with individuals experiencing mental health crises are complex and can potentially involve safety risks. There are longstanding calls for enhanced training in deploying de-escalation strategies to reduce police use of force specifically in situations involving people in mental health crises. Given the absence of an existing protocol to evaluate de-escalation competencies specific to the realm of police mental health crisis intervention, the objective of this research was to develop and validate such a tool.

**Methods:**

Development of the framework emphasized a police and community co-design approach in which the identification of core competencies was driven by stakeholder focus groups, literature reviews, and best practices in adult-based education and experiential learning models. The tool was tested and modified using live-action simulations in which police officers were instructed to respond to five unique high-intensity mental health crisis scenarios. Internal consistency, interrater reliability, and concurrent validity were examined in a series of validation studies.

**Results:**

The resultant framework, the De-escalating Persons in Crisis Competencies Tool (DePICT™), is a 14-item rater-observer competency-based assessment designed to systematically measure a trainee's demonstrated ability to safely de-escalate and respond to a person in mental health crisis using relational policing approaches.

**Discussion:**

To better meet rising mental health crisis calls, police organizations have begun to deliver specialized training to enhance the abilities of frontline officers to recognize mental health problems and safely de-escalate crises. The DePICT™ is a reliable and valid competency-based assessment tool that, when used together in scenario training applications, contributes to the measurement, training, and acquisition of essential knowledge, skills and abilities expected of modern police officers and prepares them for real-world job demands.

## Introduction

Police officers often find themselves at the forefront of responding to mental health crises and related emergencies associated with substance abuse and homelessness. Mental health crises involve instances in which a person is experiencing acute distress, disorientation, or disturbance in their cognition, emotions, or behavior that places them at risk for being harmed or harming others (e.g., suicide-related behavior, Lavoie et al., 2022). These police-public interactions are dynamic, complex, and have the potential to carry safety risks. Police officers have a vital role to play when responding to calls for service involving those in mental health crisis. Police officers are not medical professionals and cannot diagnose illness; yet it is essential that officers be able to identify cues of mental health crisis to accurately assess risk, avoid unnecessary escalations, and engage in de-escalation efforts when viable (Oliva et al., 2010).

Longstanding calls to transform police practices in responding to mental health calls stem from a series of high-profile cases of police-involved deaths of people in crisis (Engel et al., 2019), coupled with evidence that people living with a mental health illness (PLwMI) disproportionately experience applications of force in police encounters (e.g., Hallett et al., 2021; Kane et al., 2018; Kesic et al., 2013; Nicholson and Marcoux, 2018). Adding to the challenge, police officers report sustaining injuries in responding to mental health crisis calls, underscoring officer safety risks (Kesic et al., 2013). To improve police-public interactions with people in crisis, police organizations have focused, among other strategies, on developing specialized initiatives to train frontline officers to recognize mental health problems, de-escalate crises, and divert people from the criminal justice system to mental health treatment and community supports. De-escalation in this context involves strategies to reduce conflict intensity and gain cooperation with little or no application of force. Recommendations to prioritize de-escalation practices made by the President's Task Force on 21st Century Policing (2015) and echoed in landmark inquiries across additional regions [e.g., Braidwood (2010) and Office of the Chief Coroner (2024) inquiries in Canada] have made police de-escalation training common place today.

Research literature examining broad de-escalation training efforts in police contexts has grown substantially in recent years; however, more research focus is needed on minimizing use of force (UOF) in police responses to mental health crises. Recommendations guiding the development of police de-escalation initiatives in the context of mental health crisis response emphasizes researcher, mental health professional, community expert and stakeholder partnerships (Andersen, 2024; Coleman and Cotton, 2014; Iacobucci, 2014; Usher and Trueman, 2015). Central to evidence-based training is the development and routine use of valid assessment tools to measure an officer's acquired skills and abilities. Yet, few protocols exist that clearly identify or systematically assess de-escalation core competencies in the unique realm of policing, and fewer still in the specific context of mental health crisis. Consequently, the objective of this study was to co-develop and validate a competencies-based assessment tool informed by diverse perspectives of community stakeholders, including police. The current study was part of a large 4-year project supported by the Social Sciences Humanities Research Council of Canada that investigated the effectiveness of a community co-designed and delivered scenario-based training program in improving police responses to individuals in mental health crisis (Lavoie et al., 2022). Guided by the recommendations of TEMPO: Training and Education about Mental Health for Police Organizations (Coleman and Cotton, 2014), this initiative prioritized the perspectives of people with lived experience of mental illness in the building and delivery of the curriculum to realize its core objectives in reducing stigma, enhancing procedural justice, and improving the overall quality of interactions between police and individuals in mental health crisis. While we have detailed the methodology for the development of this scenario-based program previously, in this paper, we focus exclusively on the process and products of community stakeholder co-development of an associated evaluation framework. Specifically, we outline how stakeholder perspectives were synthesized into the De-escalating Persons in Crisis Competencies Tool (DePICT™), an assessment protocol that establishes core police de-escalation competencies in the specific setting of mental health crisis response. Consequently, the purpose of this paper is to describe the innovative co-development and validation methods of generating this useful tool, and to ultimately present it for accessible use in the field. A competency-based assessment such as the DePICT™ serves to support individual officer skills development in de-escalation of mental health crises and enhance professional readiness while providing a framework for program evaluation and organizational accountability.

## Literature review

In recent years, the rising prevalence of mental health-related calls for police service has become a pressing concern across the globe. Contacts between police and people in mental health crisis, while difficult to measure, are numerous relative to the general population (Huey et al., 2021a) and have continued to burgeon in jurisdictions around the world (e.g., Kane et al., 2021). This surge is attributed in large part to profound deficiencies in accessible mental health treatments and services in the community, as well as the COVID-19 pandemic's negative impacts on mental health conditions (Huey et al., 2021b; Xiong et al., 2020). Contemporary estimates suggest that mental health-related calls account for between 1% and 30% of police contacts in regions sampled across North America and Europe (Huey et al., 2021a; Kane et al., 2021; Savage and Moribito, 2022; Thomas, 2024). While most people in crisis are known to mental health services and have received past treatment for their conditions, police constitute a key pathway for about one in ten citizens not yet connected to mental health services (Dodd et al., 2024).

Indeed, police work has expanded toward responding to calls for service related to mental health and social disorder as opposed to responding to activities that are explicitly criminal in nature (Standing Committee on Public Safety National Security, 2014; Watson and El-Sabawi, 2023). In many communities, police officers remain the primary and sole emergency responders to mental health emergencies (Kane et al., 2018; Richmond and Gibbs, 2021). Mental health calls for police service can be challenging because they frequently involve citizens who have difficulty understanding and complying with police instructions (Kesic et al., 2013), are fearful or distrustful of police (Xanthopoulou et al., 2022), and are at times in possession of weapons (Dodd et al., 2024). While there are, fortunately, expanding efforts in many regions to deploy dedicated crisis intervention units [i.e., co-responder teams, Crisis Intervention Teams, non-police crisis units (Huey et al., 2021b; Wood et al., 2021)], the immense and growing unmet mental health needs within communities signal that frontline police officers will be tasked with responding to elevated crisis calls for the foreseeable future.

While the vast majority of police interactions with the public are resolved peacefully (Baldwin et al., 2018; Kiedrowski et al., 2015), some interactions between police and people in crisis result in avoidable harm. Citizens living with mental health conditions or experiencing crisis are significantly more likely be subjected to police UOF (Hallett et al., 2021; Jun et al., 2020; Kane et al., 2018; Kesic et al., 2013; Moribito et al., 2017; Rohrer, 2021). This risk increases when additional factors of identity such as race, intersect, making racialized individuals particularly vulnerable (Nicholson and Marcoux, 2018; Wortley et al., 2020; Wortley and Jung, 2020; Wortley et al., 2021). International policing research literature has robustly shown that PLwMI are disproportionately killed in police interactions across America and the Commonwealth (Australian Institute of Criminology, 2013; Fuller et al., 2015; Nicholson and Marcoux, 2018; Rohrer, 2021; Saleh et al., 2018). In addition, the rise in “suicide-by-cop” incidents, where individuals intentionally behave in a threatening manner to provoke law enforcement into using lethal force against them, is a deeply concerning component of police-involved fatalities (Isaza, 2020). Such incidents commonly feature a psychiatric element (De Similien and Okorafor, 2017; Dewey et al., 2013; Kesic et al., 2012) and may be circumvented through de-escalation efforts (Weiss, 2022).

Extending beyond concerns of disproportionate police UOF is *how* people in mental health crisis are generally treated in interactions with law enforcement. While mental health training delivery has increased among law enforcement personnel over the past two decades (Fiske et al., 2020), citizens experiencing mental health crisis continue to report feeling criminalized, devalued, and stigmatized during encounters with police (Boscarato et al., 2014; Morgan, 2024; van der Meulen et al., 2021; Xanthopoulou et al., 2022). This acknowledgement is not intended to undermine the valuable work of many officers who dedicate their best efforts in service to their communities- in fact, many PLwMI do report positive encounters with police (e.g., Brink et al., 2011)—but rather to point out opportunities for continued improvement in police training and practice. Xanthopoulou et al. (2022) systematic review of studies investigating first responder crisis interventions revealed that PLwMI describe fear, confusion, and shame during police encounters. Further, first responders held persistent beliefs that PLwMI were dangerous, unpredictable, and incapable of cooperation. Altogether, research from the perspectives of PLwMI highlights that the way police treat citizens has profound consequences in that it can influence the reactions of others the interaction and can shape the overall outcome of encounter either negatively or positively.

Both police experts and the public endorse the use of de-escalation as a preferred approach to best practice (Engel et al., 2020). Effective de-escalation training is thought to reduce police UOF and consequently bolsters public confidence and positive perceptions of the police (Stanko and Bradford, 2009). While the delivery of police de-escalation training is more common today, *there is relatively less focus on the mental health crisis context despite that these encounters continue to present significant challenges*. Ongoing training improvements to equip officers with skills to safely manage the myriad of mental health crisis situations unfolding in the community by using approaches that minimize the UOF should continue to be prioritized (Andersen et al., 2017; Coleman and Cotton, 2014; Dubé, 2016; Iacobucci, 2014; Office of the Chief Coroner, 2024).

An overview of various de-escalation training programs for police is beyond the scope of this paper having been summarized elsewhere (e.g., Engel et al., 2020); rather we refine our focus on relevant competency evaluations grounded in research. Currently, there are few tools available to assist with measuring de-escalation competencies in the policing realm, especially ones that focus on mental health crisis. Encouragingly, officers often possess the skills necessary to de-escalate and help others, but they need and deserve training to apply those skills optimally (Andersen et al., 2017; Oliva et al., 2010). In responding to situations, officers rely on the knowledge, skills, and tools in which they have been trained; as such, officers must be adequately educated to possess the requisite knowledge and trained to hone the skills and abilities necessary for operational readiness (Bennell et al., 2021; Di Nota and Huhta, 2019). Given that “the profession of policing is competency-based” (Beckley, 2004, p. 1), competency-based learning and assessment models are well-suited for their training and evaluation.

### Competencies-based approaches to assessment

Competency-based assessment (CBA) is a structured approach to evaluating an individual's core competencies, that is, the essential knowledge, attitudes, attributes, and skills required for effective job performance and readiness to meet the demands of the job (Wong, 2020). Competencies can be approached as a set of behaviors that comprise three components commonly known as “KSAs”: (i) knowledge (e.g., practical, theoretical, legislative acumen), (ii) skills (e.g., ability to perform tasks) and (iii) attitudes (e.g., personal attributes, values, ethics). CBAs involve systematic observation of an individual or a group of individuals and is designed to appraise performance against a predetermined standard (Potgieter and Van Der Merwe, 2002). CBA protocols that make use of scenario exercises such as placing learners in live or simulated contexts that mirroring real-world challenges is one way of evaluating an individual's competencies (Henri et al., 2017). This approach focuses on observing and measuring core competencies where interpersonal skills are particularly fundamental (Potgieter and Van Der Merwe, 2002). Competencies assessment using scenario-based approaches often includes hands-on simulations, problem-based tasks, and role-playing exercises that require learners to demonstrate applied elements of core competencies (Di Nota and Huhta, 2019). Conducting simulations for the purposes of CBA requires that the designers engage in careful structuring, including establishing clear learning objectives for the session, expected role-player responses, and rubrics to assess competencies (Koedijk et al., 2021; Oola et al., 2024; Potgieter and Van Der Merwe, 2002). The aim is to determine how the learner might respond to real occupational conditions and pressures. Observing performance is particularly suitable for assessing job competence in policing where officers are expected to make decisions and execute skills under stressful or high intensity conditions (Koedijk et al., 2021). Scenario-based assessment can be facilitated by establishing collaborative learning environments (Oola et al., 2024). CBAs are considered beneficial in that they are fair, objective, transparent, possess high face validity and allow for tailored and timely feedback for learners (Potgieter and Van Der Merwe, 2002).

### Current landscape in de-escalation competencies

Recent narrative and scoping literature reviews of research focused on police de-escalation have illuminated several de-escalation fundamentals. The most common KSAs consistently identified in research reviews of de-escalation practices tend to fall into eight broad categories: (1) *Strong communication skills:* Police must possess both verbal, as well as non-verbal (e.g., non-threatening paralinguistics, body language, facial expressions, proxemics) communication skills that foster de-escalation and cooperation, as well as have knowledge of impediments to communication, such as cognitive, psychological, physiological, developmental factors (Bennell et al., 2022; Huey et al., 2021b; Zaizer et al., 2023). Active listening, establishing rapport, and asking simple open-ended questions are specific examples of communication strategies that are cited frequently (van Lith et al., 2024). (2) *Conveying empathy and compassion*: Police must be proficient in offering compassion for a person's situation and demonstrating empathy for how a person feels in a moment of crisis. Acknowledging and validating citizen's emotions and experience, allowing emotional venting, removing stressors (e.g., flashing lights) and apologizing are relevant techniques (Bennell et al., 2022; van Lith et al., 2024). (3) *Respectful interactions with equity-deserving citizens*: Officers must be equipped to interact productively and respectfully with community members from equity-deserving groups, specifically citizens from diverse groups with historic or current tenuous relationships with police (e.g., racialized individuals, PLwMI, or substance use problems, LGBTQ2+ community members). Officers must have an awareness of implicit bias and its impact on their behavior, and develop competences in cultural safety (Bennell et al., 2022; Huey et al., 2021b). (4) *Procedurally just treatment:* Procedurally fair approaches, such as those that encourage involvement of the citizen in resolving the conflict, neutrality in the officer's decision-making, expressions of dignity and respect, and efforts that convey that the police are trustworthy, can generate positive outcomes such as a higher likelihood of citizen cooperation and compliance (Bennell et al., 2022; Watson and Angell, 2007; Wood and Watson, 2017). Honesty, transparency, compromise, and giving choices are specific techniques that foster procedural justice (van Lith et al., 2024). (5) *Stress and emotional self-regulation*: Police are routinely tasked with regulating their own emotions and behaviors to make sound decisions and solve problems during public interactions, which can involve high pressure, danger and uncertainty (Bennell et al., 2022; Huey et al., 2021b). Officers who can recognize their own heightened stress state, understand how stress effects their performance, and the ability to manage these effects can enhance their own performance in responding to elevated encounters (Andersen et al., 2024; Bennell et al., 2022). Specific strategies include remaining calm, not rushing, repositioning (van Lith et al., 2024), and self-regulation through conditioned adaptive breathing techniques (Andersen et al., 2024). (6) *Understanding the nature of conflict and power dynamics:* Officers require a fundamental awareness of how conflicts typically unfold, and of conflict dynamics that can escalate the volatility of the situation (Zaizer et al., 2023). Officers who know that humans tend to reciprocate the actions of another person can avoid being drawn into a “conflict spiral” (Zaizer et al., 2023). Police responding to elevated situations do well to make clear their role as officers of the peace (van Lith et al., 2024). (7) *Risk assessment and situational awareness*. Officers are in optimal positions to set the table for de-escalation when the situation is made safe for everyone. The officer's capacity to accurately perceive, plan for, and act to manage potential threats in the environment safeguards the officer, person in crisis and others (Bennell et al., 2022). Specific strategies include permitting distance, using cover, and containing risks. (8) *Attitude invested in de-escalation:* Officers who adopt a guardian mindset which prioritizes communication, trust and relationship building and community service are more committed to de-escalation practices (McLean et al., 2020). Conversely, those with warrior mindsets emphasize authoritative approaches, readiness for confrontation, and vigilance toward threat cues, which in combination tends to inflame situations (Zaizer et al., 2023). Altogether, these KSAs are a jumping off point for de-escalation training more generally that are relevant in considering de-escalation in the specific context of mental health crisis.

To augment this constellation of competencies, there has been some attention on determining key KSAs for police de-escalation in situations involving mental health crisis, specifically. Many researchers contend that a deeper understanding of mental health issues, including the ability to recognize and respond effectively to signs and symptoms of mental illness or crisis is paramount to safe de-escalation practices with this population, as is the ability to engage in informed and accurate, risk assessment (Cotton and Coleman, 2010; Coleman and Cotton, 2014; Lavoie et al., 2022; Oliva et al., 2010). Further, an awareness and reduction of stigma directed toward PLwMI is vital to police education, in particular addressing myths concerning the relationship between mental illness and violence (Coleman and Cotton, 2014). Knowledge of laws (authorities that govern the UOF), contextually relevant legislation (e.g., mental health acts), and organizational polices (e.g., transfer of custody at hospital, procedures for summoning co-responder units) is foundational to effective de-escalation of PLwMI (Bennell et al., 2022; Huey et al., 2021b), as is familiarity with community mental health resources and services to bolster referrals and make alternative options for resolution available (Bennell et al., 2022; Coleman and Cotton, 2014; Huey et al., 2021b; Lavoie et al., 2022).

Altogether, there are longstanding calls for improved training in the application of de-escalation practices to mitigate police UOF particularly in encounters involving individuals experiencing mental health crises. In response to the increasing volume of mental health-related service calls, police agencies have implemented specialized training programs aimed at enhancing frontline officers' capacities to identify mental health conditions, de-escalate crises safely, and facilitate diversion from the criminal justice system toward more appropriate services. Training programs are well-served by relying on fair and valid CBA tools to measure program effectiveness and evaluate individual officer acquisition of targeted skill sets. However, there is a paucity of existing protocols to assess de-escalation competencies specific to the realm of police mental health crisis intervention. Consequently, the goal of this research was to develop and validate such a tool.

## Methods

### Community co-conceptualization of de-escalation competencies in MH crisis response

While there is an abundance of scenario-based training in policing contexts, our study identified a need within the scholarly literature and independent reviews for both evidence-based training in de-escalation and mental health crisis response and training that prioritizes the perspectives of PLwMI alongside clinicians and community advocates (Coleman and Cotton, 2014; Iacobucci, 2014; Usher and Trueman, 2015). Partnerships between university research teams, the community, and police are necessary to assist police services in developing robust, evidence-based, and community-responsive training (Iacobucci, 2014). The complexity of mental health crisis calls exceeds the capacity and expertise of any single vantage point or disciplinary perspective, requiring a transdisciplinary approach to training design and delivery that includes PLwMI. Such individuals who have had encounters with police in the context of a mental health crisis are best positioned to participate in the determination of de-escalation competencies since they have first-hand knowledge of what effective and in-effective de-escalation efforts “looks like and feels like.”

The 4-year study period allowed relationships of mutual respect and trust to form over an extended development process (Bland and Epstein, 2008), as well as compensation for the contributions and commitments of community members. The research team driving the co-design process was led by a group of three co-investigators who guided the work of 12 core collaborators composed of people with lived experience; community advocates; clinicians; police chiefs, trainers, and front-line officers with specialization in mental health crisis response; and researchers with expertise in clinical psychology, cultural safety, intercultural communication, simulation-based training and evaluation, and adult education. The diversity of the team supported a prismatic and holistic approach to the broader study, which aimed to investigate whether scenario-based training could improve police response to individuals in mental health crisis and, if so, how. The goal of the current study was to detail the co-design processes underlying the construction of the evaluation framework that captures the core competencies of police de-escalation in the specific context of responding to mental health calls for service. The authors have elsewhere detailed the methodology for the development, delivery, and testing of the 40-h, scenario-based program guided by the core competencies in de-escalation identified in the DePICT™ (Lavoie et al., 2022). At the outset of the study, the research team identified the need for an evaluation framework that was valid, evidence-based, and composed of a comprehensive set of core competencies that were consistent with mandated training in basic constable courses. Moreover, the evaluation framework needed to be designed by a diverse group of stakeholders to ensure the tool included a comprehensive set of competencies. Competencies needed to be identified in partnership with those who have first-hand insights into effective police response to mental health crisis based on their lived experiences alongside those who have the tactical knowledge to identify the skills necessary to maintain the safety of all parties involved without recourse to the UOF. The research team identified that this integration of tactics in effective de-escalation strategies was paramount. Importantly, the inclusion of de-escalation trainers in the design of the assessment tool as end-users allowed the team to iteratively test the tool to ensure it remained reliable and accessible for trainers to use.

### Stages of instrument development

Part of a 4-year, SSHRC-funded study, the development of the DePICT™ was conducted in six phases: (1) assessment context and parameters of use; (2) literature review and landscape analysis; (3) focus group discussions for indicator development; (4) scenario creation for competencies assessment; (5) instrument design; and (6) field testing and validation. This phased process aligned with recommended practices for the development of observational behavior assessment tools in the context of competency-based police training (Koedijk et al., 2021). Delphi techniques were also adapted to guide an iterative development process that relied on collecting, distilling, and refining the input of subject matter experts and seeking consensus among a diversity of perspectives (Wong, 2020). A description of the team formation and partnership building process can be found in Lavoie et al. (2022).

### Phase 1: establishing the assessment context and parameters

In its first meetings, the research team established a shared understanding of the assessment context and parameters. Following the recommendations of key reports from across Ontario, Canada, which identified the need for a valid police de-escalation assessment framework that could be used in the context of mental health crisis response, the team confirmed that the evaluation of competencies should move beyond simple “pass or fail” outcomes (e.g., Dubé, 2016; Iacobucci, 2014; Di Nota et al., 2021b), a perspective that is also widely supported in police research (Di Nota et al., 2021a). Instead, assessment should provide formative learning opportunities that support a growth mindset in officers and reinforce the continuous enhancement of de-escalation competencies as an integral part of their professional practice. The research team noted the importance of identifying core competencies that would be applicable to a range of mental health crisis encounters and require demonstrable proficiencies by officers at all levels from recruit to senior. The assessment tool was designed for scenario-based training contexts that aim to simulate high fidelity, dynamic encounters across a diversity of mental health crises that officers commonly encounter on the road. The research team identified the importance of creating scenario-based assessment contexts in which officers have repeated opportunities for “rehearsal learning” to deepen the acquisition of competencies through embodied practice while enhancing the capacity for ethical decision-making under stress (Koedijk et al., 2021; Di Nota and Huhta, 2019; Smith et al., 2016). While the tool was designed primarily for scenario-based training contexts, the tool has been shared with researchers internationally for use in real-world settings to examine officers' demonstration of de-escalation competencies in mental health crisis encounters with members of the public.

### Phase 2: literature review and landscape analysis

A literature review and landscape analysis were conducted to acquire a comprehensive understanding of police training objectives, existing training legislation, recommendations emerging from inquest reports on mental health crisis encounters involving lethal force, and de-escalation assessment frameworks in neighboring fields of practice. To amplify the perspectives of team members who have experienced mental health crisis encounters with police, literature on consumer perspectives (e.g., Livingston et al., 2014) and procedural justice (e.g., Watson et al., 2010) was examined to identify optimal officer behaviors and decision-making based on consumer reflections and preferences. Research literature on de-escalation in clinical psychiatric settings was also reviewed. While no de-escalation evaluation tools specific to mental health crisis intervention in the policing context were identified, the team analyzed comparable competency-based frameworks, such as the Harvard University (2008) used in performance management processes and the seven-item De-escalating Aggressive Behavior Scale—English Modified Version (EM-DABS) developed to assess the performance of psychiatric nursing students in managing aggressive behavior in patients (Mavandadi et al., 2016). The key competencies that comprise the Constable Competency Profile created by the Government of Canada's Police Sector Council were also examined to ensure alignment with the proficiencies expected of the general policing role at the rank of constable (Police Sector Council, 2009). The literature review and landscape analysis allowed the research team to identify areas of focus that guided the next phases of indicator development and scenario creation.

### Phase 3: focus group discussions for item generation

Phase 3 focused on the identification of core competencies required for effective de-escalation in mental health crisis response. The research team was divided into focus groups composed of 4–5 subject matter experts with diverse disciplinary and lived perspectives (e.g., PLwMI, clinicians, police instructors, researchers). Each group was given 30 min and materials including markers, large Post-it paper, and an easel to discuss and brainstorm a list of 5–8 competencies necessary to respond effectively to a mental health crisis. The groups then came together to share the competencies they had generated. The group conducted a thematic analysis of group-generated competencies to identify emergent areas of significance. From this exercise, the group arrived at seven broad competency domains: communication, risk assessment; self-awareness and self-regulation; judgment and decision-making; sensitivity; procedural justice; and ethical accountability. The group was then tasked with identifying the specific behavioral skills that comprise each broad competency domain, that is, the observable KSAs officers would be required to demonstrate in a scenario. An initial set of 37 indicators was generated by the group (~6/domain). The list of indicators was then analyzed as a group, focusing on repetition across domains and whether competencies were indeed observable and, if so, how (e.g., how might an officer demonstrate self-awareness and self-regulation?). In keeping with recommendations made by Koedijk et al. (2021), the list of the indicators was used as a basis for scenario design in the next phase.

A key finding at this stage of development was the identification of “Relational Policing” as a key component underlying competencies in the de-escalation of people in mental health crisis. At its heart, relational policing motivates officers to reflect inwards about what they bring to an encounter as an individual with potential biases and preconceptions and as a person in uniform (Álvarez, 2021). When engaging “relationally,” officers are reflective about their authoritative positioning and recognize that they are co-implicated in the encounter out of a sense of responsibility for the person's welfare and wellbeing with the knowledge that no one (including themselves) is invulnerable to mental health crisis (Lavoie et al., 2022). This self-reflection fosters genuine, personal, and empathetic police responses to issues of public safety. Relational policing involves a number of interconnected response methods available to officers engaging in public-police interactions, including: humanized, person-centered, procedurally just, empathic, trauma-informed, mental health and crisis aware, and culturally safe approaches (see Lavoie et al., 2022).

### Phase 4: scenario creation for competencies assessment

In this 4th phase, the research team turned to scenario creation to support the acquisition and assessment of de-escalation of mental health crisis competencies. Ensuring a balance of diverse perspectives and areas of specialization, the research team was once again assembled into small focus groups to design scenarios that would provide opportunities for officers to demonstrate the identified competencies. The team stressed the importance of creating scenarios based on commonly encountered mental health crises to maintain realism and ecological validity, thereby ensuring the scenarios have “predictive value for behavior in daily reality” (Koedijk et al., 2021, p. 4; Di Nota et al., 2021a). Each group was given 45 min to develop a scenario outline in response to guiding questions: (1) Who is the person in mental health crisis (e.g. race, class, gender)?; (2) Where are we and what is the context?; (3) How is the person presenting? What behavior or actions are bringing the police to the location? Is the individual presenting an immediate or imminent threat to themselves or others?; (4) What triggered the situation? What circumstances or personal history has led to this moment?; (5) Who else is present? How are others reacting or responding to the person in mental health crisis? How are they reacting to the present of police?; and (6) What do you hope the trainee would do to engage effectively with the individual in mental health crisis? The research team reconvened to share the scenario outlines each group had developed, discussing optimal officer responses in each context and cross-checking these optimal responses against the group's existing list of indicators. This exercise allowed the group to further refine the list of indicators by identifying gaps as well as expected levels of competence among recruits and more experienced officers. Competency mapping in the context of scenarios depicting commonly encountered mental health followed best practices in competencies development anchored in rigorous environmental and job analysis that include the perspectives of stakeholders and subject matter experts (Campion et al., 2011).

### Phase 5: item refinement and instrument design

For phase 5 of the methodology, a subcommittee of five team members was struck to focus on instrument design. As a first step, the team organized the complete list of indicators into thematic groupings according to general competency areas. Close analysis revealed further areas of duplication and overlap, which allowed the initial list of 37 indicators to be distilled to an essential list of 14 core competencies. The team determined that quantitative indicators would support a pass/fail threshold to establish a professional standard of successful completion while providing the ability to score indicators on a spectrum and foster an effective debriefing tool for instructors to readily reinforce competency areas of strength and identify weakness for enhancement as demonstrated by trainees. A rater-observer checklist was created in keeping with familiar methods of scenario-based assessment in police training contexts in which instructors observe a trainee's performance, knowledge, and skills (Bondarenko et al., 2020; Koedijk et al., 2021). The scoring method followed Likert-anchored response options, which initially followed a three-point scale of 2 = excellent, 1 = needs improvement, and 0 = poor based on the degree and frequency of each skill demonstrated by the trainee. After field testing (see Phase 6), the scoring method was developed into a 4-point scale to support more exacting measurement based on observable indications of each competency during the scenario assessment, from 3 = Proficient, 2 = Satisfactory, 1 = Emerging, to 0 = Absent. The subcommittee shared the 14-item checklist with the broader research team for feedback prior to field testing and elected to name the tool as the De-escalating Persons in Crisis Competencies Tool (DePICT™).

### Phase 6: field testing and validation

In phase 6, eight scenarios created by the group were taken through a rehearsal process with professional actors and staged for the research team to ensure they created the necessary conditions for an officer's demonstration of de-escalating a mental health crisis. The scenarios were assessed by the research team for authenticity with respect to the representation of mental health crisis behaviors as well as environmental factors, including set configurations and proxemics. This holistic evaluation of scenarios by a diverse team of subject matter experts ensured that they served as effective training scenarios, providing officers with the opportunity to integrate tactical considerations, set the table for safe and effective de-escalation, identify signs and symptoms of mental health crisis, and conduct accurate risk assessments.

Using volunteer officers ranging in experience from constable to staff sergeant, the scenarios were staged once again with the research team to conduct field-testing with the most recent iteration of the rater-observer checklist. The research team observed the officers as they engaged in each scenario and coded the checklist *in situ* as the scenario unfolded. A group discussion followed in which team members shared their scores item by item to identify points of divergence and consistency in their determinations of what differentiates a competency scored at a level of “Proficient” (3), for example, from a “Satisfactory” (2). These discussions led to further clarification of indicator definitions, which formed the basis of the coding manual instructions.

In partnership with a police service in southern Ontario, the DePICT™ was field tested across three waves of data collection with a total of 71 frontline police officers comprising both recruits (38%) and experienced patrol constables (62%). Years of service among the sample ranged from less than a year to 29 years. With respect to participant demographic characteristics, the mean age was 30.48 years (*SD* = 6.75). Most officers were male (62.9%; 37.1% female) and identified as White (72.9%; 12.9% Black). Officers were recruited through volunteer sampling, a non-probability sampling method where researchers rely on individuals who express interest in joining the study based on advertisement through training instructors at the police service to receive a week of mental health crisis response training. The research team aimed to recruit 24 officers for each wave of data collection and were successful in meeting this target, with minor expected attrition due to illness. Data collection took place in an unoccupied wing of a local retirement facility which featured separate apartments that were intentionally staged for each scenario. Officers participated individually in a circuit of five unique scenarios featuring professional actors with specialized training who, following rehearsal preparation, are experts in embodying specific symptoms as well as improvising responses to meet specific scenario objectives. Safety officers were briefed and attended at every station. Each simulation was 10-min in length and introduced demographically diverse individuals experiencing a variety of mental health crises (e.g., suicide-related, active symptoms of psychosis) of varying intensity.

In the first wave of the study, 24 officer participants took part in each of the five scenarios which were video recorded for later analysis; the performance of each participant was consensus-coded in real time by a pair of expert raters from the research team (i.e., 1 police instructor and 1 of cultural safety expert, forensic psychologist, psychiatric nurse, or criminologist). This initial wave of field testing resulted in a restructure of item order in the instrument: indicators previously organized according to thematic groupings based on competency domain (e.g., communication, procedural justice) were re-organized according to a sequence of officer actions that better reflect the typical progression of crisis encounters. Team members observed that an officer's first steps predictably entail assessing and managing safety considerations, followed by engaging in demonstrable efforts to lower the intensity of the crisis through communication and reassurance of help, prior to moving into collaborative approaches to determine steps toward resolution. This re-organization of indicators resulted in a more user-friendly tool that follows the sequence of competencies trainers are looking for as the scenario unfolds. Additionally, it elucidates the effect a poor approach an initial contact can have on the subsequent competencies.

The restructured version of the DePICT™ was field tested in two additional waves: a second study sample of 23 frontline officers followed by a third study sample of 24 frontline officers all from the same mid-size police service in southern Ontario, which serves multiple communities with a combined population of 700,000 residents; in 2022, mental health crisis calls represented ~5,000 of its ~150,000 annual calls for service. Each study group moved through the same circuit of five scenarios with actors where their performance was recorded. Two assessors once again used consensus-coding methods to score the DePICT™ for each participant. The results of these additional waves of field testing led to a careful revision of the training manual and competency operationalizations to reduce existing competency overlap and arrive at mutually exclusive indicators. In accordance with Wong (2020), the training manual was further developed to provide clear definitions of each competency, a description of observable behavioral indicators officers may demonstrate at the highest level of proficiency, along with clear coding instructions. A 45-min training video with scenario vignettes was produced for police trainers, allowing trainers to practice coding and develop a confident command of the DePICT™ evaluation method. The DePICT™ was trademarked by the research team to dissuade unapproved changes to the framework when used in the field by external police agencies. This step serves to foster version control and consistency in establishing an evidence base in measuring competencies in police officer de-escalation of mental health crisis.

Factor structure, internal consistency, interrater reliability, and concurrent validity were examined in a series of validation studies. All analyses were performed using SPSS Version 27. Principal Components Analysis (PCA) was used to estimate the underlying structure of the tool by considering DePICT™ item scores rated by consensus from the 47 unique participants who took part in waves 2 and 3 of the field study. Item scores across all five scenarios were used in the analysis to represent DePICT™ performance across various levels of scenario intensity (i.e., 235 sets of item scores). The model estimated using the covariance matrix extracted a single dimension suggesting a univariate structure (i.e., 1 component with an eigenvalue above 1.00 confirmed by visual inspection of the Scree Plot, all items loading onto a singular component above 0.400). The model accounted for 49.4% of the variance in the data. Psychometric testing of the same data revealed that the DePICT™ possesses excellent internal consistency (Cronbach's α = 0.914, *N* = 14 items) indicating superior scale reliability. No floor or ceiling effects were identified as determined by obtaining the percentage of participants reaching the lowest and highest possible scores (lowest score = 0%, highest score < 1%). Concurrent validity testing of the DePICT™ was undertaken with the English Modified De-escalating Aggressive Behavior Scale (EmDABS, Mavandadi et al., 2016), a psychometrically validated tool designed to measure de-escalation in the psychiatric setting. Analyses relied on individually rating available video footage of *N* = 101 simulations that were previously recorded from previous field testing. Two criminology graduate students were trained to code the EmDABS (Cronbach's α = 0.854; Inter-ICC = 0.749) and then rated the videoed scenario performances to compare ratings with DePICT™ total scores arrived at by consensus for the same scenarios. A Pearson Correlation Coefficient was used to estimate concurrent validity, given that both scale total scores were continuous and normally distributed (Kolmogorov-Smirnov Goodness of Fit Test = *ns*). Results indicated a “good” level of association, *r* = 0.68, *p* = 0.0001, *N* = 101 (Intra-rater, ICC = 0.713), supporting that the DePICT™ has concurrent validity with a similar tool used to measure de-escalation of people in mental health crisis. Additional interrater reliability testing was established in a separate study and can be found in Lavoie et al. (2023).

As a further step to validation, the DePICT™ was presented to a large community of practice composed of ~100 police instructors across Ontario police services. The DePICT™ received positive feedback regarding utility and feasibility. Six representatives from San'yas Indigenous Cultural Safety in Ontario and British Columbia conducted a further review of the tool and commended its overall alignment with core competencies in cultural safety. In 2019, the DePICT™ was licensed to Ontario's Ministry of the Solicitor General (formerly the Ministry of Community Safety and Correctional Services) for use at the Ontario Police College.

In sum, the 6-phase instrument development process used multiple data collection methods in iterative cycles of competencies development and competencies assessment (Wong, 2020) to arrive at a stakeholder-driven, user-friendly, and scientifically validated tool specific to policing and mental health crisis response. While the DePICT™ is an evidence-based tool to guide micro-level training objectives, providing clear guidance to trainees on strengths and areas in need of improvement to support their career pathway, it may also be used to support macro-level strategic planning in the establishment of training guidelines, curricular outcomes, and professional standards in policing and mental health crisis response.

## Results

The resultant framework, the De-escalating Persons in Crisis Competencies Tool (DePICT™), is a 14-item rater-observer CBA designed to systematically measure a trainee's demonstrated ability to safely de-escalate and respond to a person in mental health crisis using relational policing approaches. Final competencies and accompanying definitions are presented in Table 1.

**Table 1:** DePICT™: De-escalating Persons in Crisis Competencies Tool (2025) item descriptions, CC BY-NC 4.0.

**No**.	**Item**	**Definition**
1	Approaches, contains, and controls the scene for effective risk management	Officers accurately identify the scope of risk to safety, threat cues, and imminence, and manage these accordingly. Officers scan and assess the scene for safety risks at initial approach and throughout the encounter. Officers contain and control elements of the scene to enhance safety and proactively manage potential threats to reduce the need to respond urgently. Officers approach and control the situation to create an environment that is safe for every person (i.e., themselves, person in crisis, other first responders, and public).
2	Manages time and distance	When appropriate, officers slow down the pace of the encounter. Officers take their time and create distance to decrease imminence or the intensity of the situation and permit a reactionary gap to manage perceived threats. Officers use cover and barriers as necessary to enhance protection and create a safe cushion of space.
3	Expresses genuine concern for welfare and willingness to help	Officers demonstrate genuine concern for the welfare of the person in crisis and convey a willingness to assist them. Officers offer reassurance that they are there to help. Officers act as an ally. Officers accept, rather than dismiss, the concerns of the individual and assure them that their concerns will be addressed.
4	Humanizes connection and promotes dignity	Officers humanize themselves and convey that they view the person in crisis as a person of value. Officers personalize the encounter and offer understanding and compassion about the person's situation. Officers use a relational approach to make a human connection and build rapport. Officers promote the person's dignity, engage in face-saving behaviours, and work to preserve the person's self-worth.
5	Employs calming paralanguage	Officers use paralanguage (e.g., tone, pitch, pace, volume) as appropriate to minimize the person's distress, encourage communication, and convey interest in the person's situation.
6	Uses inclusive, non-judgemental and respectful language	Officers consistently use language that is respectful of the person's lived experience, culture and rights. Officers refrain from disrespectful labels or messaging. Officers are polite and courteous. Officers avoid judgmental language.
7	Exhibits calming body language and self-regulation	As appropriate, officers demonstrate posture and body language that models calm. Officers remain behaviorally calm and in control of their physiological arousal and take steps as necessary to regulate self-arousal levels. Officers refrain from using unnecessarily threatening body language (e.g., looming, blading or fighting stance, clenching fists, drawn weapon).
8	Demonstrates self-awareness and flexibility	Officers recognize when their presence, communication, or behaviour is unproductive or distressing for the person in crisis and adjust accordingly. Officers respond in ways that are trauma-informed. Officers minimize their authoritative presence as needed to reduce fear/tension. Officers continue to be responsive to the person's feedback, changing circumstances, and new information. Officers are flexible in their intervention and try new approaches when initial attempts fail.
9	Actively listens and permits emotional expression	Officers demonstrate that they are actively listening to the person for cues on how to resolve the crisis. Officers prompt and gain information to understand the situation better. Officers allow the person in crisis to express emotion and permit human expression of their experience without unnecessarily curtailing it.
10	Identifies signs and adapts response to mental health crisis behaviors	Officers inquire about and discern key signs/symptoms of mental illness or mental health crisis. Officers modify their engagement with the person in crisis based on these observed signs to foster communication and effective intervention.
11	Demonstrates validation of person's emotions and experience	Officers acknowledge and accept that the other person has valid emotions and experiences, even if these differ from the officer. Officers identify, recognize, or share the person's feelings and experiences (i.e., empathize with the person).
12	Seeks additional information and uses available resources	Officers obtain the information needed to effectively manage the crisis and generate alternate solutions throughout the encounter from available parties. Officers confirm the accuracy of dispatch/computer aided dispatch information with different information sources. Officers seek and use external input from family members, others on scene, health and social services, etc. to obtain a fulsome and accurate understanding of the situation. Officers seek assistance from and refer to available local resources and request specialized units as needed.
13	Fosters collaborative, person-centered response	Officers allow the person in crisis to inform decisions, approach, and resolution. Officers actively seek the individual's input and involve them in generating choices and informing decisions. Officers determine the values and preferences of the person and work collaboratively towards a solution to the crisis. Officers view the person as an expert in their own mental health and well-being.
14	Engages in clear and transparent decision-making	Officers convey processes to the person in a clear, truthful, and transparent way. Officers clearly explain what they are doing and why. Once officers have considered the available information, they explain why a course of action is necessary and address any raised concerns. Officers explain step-by-step what will happen next.

Contact mhcr@wlu.ca or the first author to access to the official checklist, full scoring instructions, and coding manual resources.

Each of these competencies is scored on a 4-point scale based on the degree and frequency that each competency has been demonstrated by the trainee during a scenario-based evaluation. The trainer notes examples of the competency exhibited by the trainee and selects a score reflecting the level of proficiency of that best describes the trainee's performance. Ratings are based on what the trainer observes the trainee to do or say during the evaluation scenario. Items scores range from Absent (0), Emerging (1), Satisfactory (2) to Proficient (3).

A total score can be derived by adding up all item scores where the highest possible score (42) indicates the topmost levels of de-escalation of mental health crisis competence. Functionally, the DePICT™ can be used as a tool of articulation of police de-escalation practices, as well as guide tailored debriefs for individual officers in training to identify distinct competencies for improvement or areas of mastery for reinforcement. The total score can be used to demonstrate improvement in de-escalation skills acquisition within the same trainee over time to ensure job readiness, support program evaluation, and foster organizational accountability.

## Discussion

There is a recognized need to improve police responses to heightened encounters involving people in mental health crisis while minimizing the UOF and enhancing community-police trust through continuing to prioritize de-escalation efforts to reach safe and peaceful resolutions. A specific population implicated in police reforms around de-escalation are those experiencing mental health crisis—these citizens are disproportionately subject to the UOF, and report that their experiences with police could be better if they were less stigmatizing, de-valuing and criminalizing. These concerns, therefore, present clear target areas for improvement in police training and practices. Recognizing that police training specific to this population is necessary (e.g., Iacobucci, 2014; President's Task Force on 21st Century Policing, 2015), many police organizations have been responsive and stepped-up efforts to develop and deliver training focused on mental health awareness, crisis intervention, and de-escalation strategies.

Despite this commitment to reform, the evidence measuring the effectiveness of these programs is scant or inconclusive at present (though results hint that police are heading in the right direction; Cotton and Coleman, 2010; Engel et al., 2020; Nicholson et al., 2025). The barrier to conclusively establishing “what works” is the lack of consistency in curriculum content, delivery method, and outcome measurements of success. Evidence-based training programs are informed by valid, consistent assessment tools to evaluate individual officer acquisition of core competencies and substantiate program effectiveness. The development and validation of the De-escalating Persons in Crisis Competencies Tool (DePICT™) addresses these gaps and the calls by accumulating inquests recommending standardized training and evaluation of police officer de-escalation in responses to PLwMI. This tool represents a significant contribution given that, to our knowledge, there are no other assessment tools that evaluate de-escalation in the context of people in crisis in the unique realms of law enforcement—the DePICT™ is the first of its kind.

Officers rely on their education and training to ensure they are prepared to respond to situations they are dispatched to resolve (Bennell et al., 2021). Therefore, officers must be trained in specific competencies to achieve professional readiness in responding to people experiencing elevated mental health crises. A hallmark of the competencies-based framework presented here is that it was generated using a systematic phased method that was deeply informed by a community co-design approach. The DePICT™ was constructed in consultation with a multi-perspective subject matter expert group, including people with lived experience and mental health advocates, clinicians and nurses, forensic psychologists, cultural safety experts, scholars, police leaders, and a large community of practice of UOF and defensive tactics experts.

Co-design refers to “an approach to designing with, not for” (McKercher, 2020) the people who stand to be most impacted by the work, and this approach is particularly critical in the context of police training, which has direct impacts on community safety and wellbeing. Co-design approaches that collaboratively occur over time with community stakeholders is a distinctly different approach to consultative approaches in which community input is sought after decisions have been made by a select group of “experts” who have undertaken the conceptualization and design independently. Co-design approaches are increasingly seen as the “gold standard” for public sector initiatives (Tindall et al., 2021) and while there are significant benefits to community co-design approaches—such as increasing the relevance, responsiveness, quality, and legitimacy of the work undertaken—co-design approaches do come with risks. Strategies must be undertaken to actively address the power differentials in the collaborative process among those accustomed to working in hierarchical arenas of practice such as policing, universities, and health care where definitions of who constitutes an “expert” can be quite narrow. A horizontal, collaborative approach in which the perspectives and contributions of each team member are welcomed and valued, regardless of their title, role, or affiliation, must be established at the outset. Transdisciplinary co-design approaches also involve, by necessity, team members with different vocabularies, stemming from their social location and fields of practice, and vantage points that can be, at times, opposing, leading to heated discussions and debates. Time is needed for mutual understanding to deepen alongside strong facilitation of the co-design process to ensure that mutual respect is actively fostered, and valuable team members are not inadvertently marginalized.

Focus group sessions provided a context for these stakeholders to collaborate on optimal competencies that a frontline police officer should possess when responding to a person in mental health crisis, informing what “right looks like” from diverse perspectives. Development was further pedagogically and theoretically driven by best practices in adult-based education. Experiential learning models were innovatively adapted from Forum Theatre, originally conceived by Brazilian theatre director Boal (1979). Forum Theatre served as a method of collective problem-solving in which a challenging scenario is staged, discussed, and participants-as-witnesses were invited to step into the scenario to practice alternative strategies toward a peaceful resolution. Drawing from this method, the research team developed a training model anchored in applied, realistic experiential learning through carefully designed immersive scenarios featuring a series of high-fidelity mental health crisis situations that allowed learners to practice competencies in the stress of the encounter and receive targeted feedback from subject matter experts (Lavoie et al., 2022).

The DePICT™ comprises a 14-item rater-observer assessment checklist that measures an officer's demonstrated ability to de-escalate a person in crisis during a standardized training scenario. Optimized competencies captured on the tool include items corresponding to *communication* (i.e., paralanguage, respectful language, body language, active-listening); *relational policing approaches* (e.g., empathy, personalized, dignity, validation); *mental health awareness* (i.e., identifying signs of mental illness/crisis, using appropriate resources), *person-centered approaches* (e.g., genuine concern for welfare, information gathering, collaboration, transparency); *self-awareness and regulation* (i.e., bias-awareness, trauma-informed, flexible); and, *risk assessment and management* (i.e., approach, contain and control, time and distance).

The tool provides a consistent and intuitive method for evaluating the presence of key competencies corresponding to de-escalation and relational policing approaches in response to a person in crisis, from first contact to resolution of the encounter. The DePICT™ was tested and iteratively revised using simulations in which police officers were asked to respond to a series of varying-intensity mental health crisis scenarios. Our research has demonstrated that this tool possesses good psychometric properties and upon examination, was a valid and reliable real-world framework for assessing de-escalation skills to those in crisis in law-enforcement training.

### Need for responsible use of this tool

Ethical standards require the selection of assessment techniques and tools that are reliable and valid, as well as suitable for use with the population being assessed. Our results indicate that the DePICT™ is psychometrically sound and appropriate for use within law enforcement. It is important to consider, however, *how* the tool will be used in this arena. In other disciplines, there are guidelines that describe the responsible use of tools and assessment practices (e.g., American Psychological Association, 2001; Canadian Psychological Association, 2017). We believe it is appropriate to borrow some of the concepts described in these guidelines when considering the use of the DePICT™. For example, users of this tool need to administer and interpret the DePICT™ appropriately and accurately which includes having adequate knowledge of the manual and comfort in assessing each item. We believe that the DePICT™ will primarily be used by trainers in policing, corrections, and related fields to evaluate recruit and frontline officers' competencies in de-escalating and responding to individuals in mental health crisis during training scenarios or in real time encounters. However, it is possible that the recorded results of this tool could be used in ways that have serious impacts on police officers' careers (e.g., in a Special Investigations Unit case or Department of Justice investigation) and therefore the user of the tools must have the professional competence and qualifications to responsibly assess and interpret the results of the DePICT™ assessment.

### Limitations and future directions

No research is without limitations. As such, it is important to note that this tool was developed and validated in Ontario, Canada and therefore may require additional review before it is applied in other jurisdictions due to variations in laws, police policies and authorities and mental health legislation. Further, mental health is deeply embedded in socio-cultural contexts which locally shape the perceptions, experience, attitudes, resources, and help-seeking behaviors concerning mental health problems. Consideration for these impacts must be made to ensure that the DePICT™ is an appropriate assessment framework for the community. Caution should be used in generalizing the results of the study to other populations. Because a non-probability sampling method was used to test psychometric properties of the DePICT™, the sample was not randomly selected and may not accurately represent the larger population of officers. While the sample did demographically represent characteristics of police officers in the province of Ontario, Canada, smaller samples are known to reduce validity. Further, the sample comprised officers willing to volunteer for mental health crisis training and study participation, and many were presumably motivated because they were encouraged as recruits to supplement their scenario-training experience. While the DePICT™ has been shown to be useful in training settings, the future utility and evolution of the tool must be realized by observing real-world police responses to elevated crisis situations involving PLwMI that both result in a peaceful resolution (and those that do not) and evaluating these encounters through the lens of DePICT™ framework. The prolific use of body worn cameras by law enforcement officers makes this approach viable. Such studies would be beneficial in further validating (or invalidating) these competencies deemed vital by community stakeholders, reveal new competencies, and identifying the relative importance of each competency to inform better training.

### Conclusion

Reliance on police officers as mental health interventionists is likely to continue in the face of deficiencies in community-level health systems and social resources needed to address unmet needs, prevent crises, and achieve long term outcomes; expectations about the reasonable effectiveness of police responses to mental health crises should be considered given these broader system challenges (Wood et al., 2021). Police interactions with people experiencing mental health crises are complex, require reduced UOF, and more emphasis on relational policing approaches and de-escalation. De-escalation techniques serve as a valuable intervention tool that not only benefits people who are in crisis, but also lowers police injury and liability (Oliva et al., 2010). Using a police and community co-design approach, the De-escalating Persons in Crisis Competencies Tool (DePICT™) attends to demands for diverse perspectives from police and non-police expertise to establish key officer de-escalation competencies. The DePICT™ encapsulates the tenants of relational policing (Lavoie et al., 2022) and when distilled in police practice, will continue to nurture positive encounters and build community trust. When used in scenario-based training applications, this valid and reliable tool contributes to officers' acquisition of core knowledge, skills and abilities expected of modern police officers to prepare them for real-world job demands. As a validated evaluation framework, the DePICT™ also supports macro-level strategic planning with respect to the establishment of training guidelines, curricular outcomes, and policing standards in mental health crisis response. The DePICT™ fosters a paradigm shift in which mutual understanding and cooperation between the public and police contributes to the establishment of a deeply needed performance standard in this area. The standardization of de-escalation competencies contributes to a more consistent quality of police service across communities. To move forward, police governance at the highest levels must champion the priority of de-escalation in police-public interactions. Only through this commitment can de-escalation and relational policing practices be meaningfully integrated into police policies and professional standards to drive real and lasting police reform.

## Data Availability

The original contributions presented in the study are included in the article/supplementary material, further inquiries can be directed to the corresponding author.
